# Multidisciplinary residential program for the treatment of obesity: how body composition assessed by DXA and blood chemistry parameters change during hospitalization and which variations in body composition occur from discharge up to 1-year follow-up

**DOI:** 10.1007/s40519-022-01412-8

**Published:** 2022-06-01

**Authors:** Clara Gasparri, Simone Perna, Gabriella Peroni, Antonella Riva, Giovanna Petrangolini, Milena Anna Faliva, Maurizio Naso, Mariangela Rondanelli

**Affiliations:** 1 Endocrinology and Nutrition Unit, Azienda di Servizi alla Persona ‘‘Istituto Santa Margherita’’, University of Pavia, 27100 Pavia, Italy; 2 Department of Biology, College of Science, University of Bahrain, Sakhir Campus, P.O. Box 32038, Zallaq, Kingdom of Bahrain; 3 Research and Development Unit, Indena, 20139 Milan, Italy; 4 Department of Public Health, Experimental and Forensic Medicine, University of Pavia, 27100 Pavia, Italy; 5 IRCCS Mondino Foundation, 27100 Pavia, Italy

**Keywords:** Obesity, Multidisciplinary residential program, Hospitalization, Body composition

## Abstract

**Purpose:**

Obesity is a chronic disease characterized by a complex variable clinical presentation with comorbidities. A multidisciplinary residential program (MRP) represents one of the best options for treating obesity. The purpose of this study was to evaluate the effectiveness of 8-week MRP on weight loss, body composition assessed by DXA, and metabolic blood parameters between entry (T0) and discharge (T1). The secondary endpoint was the evaluation of the patients' adherence to diet during the check-up outpatient visits, at 2 (T2), 6 (T3), and 12 (T4) months after discharge.

**Methods:**

168 subjects were enrolled (61 males and 117 females, aged 58.5 ± 13 years, BMI 41.3 ± 6 kg/m^2^) in the study. The difference in values ​​(end of hospitalization compared to baseline) was calculated through the univariate analysis procedure, which provides regression analysis and analysis of variance for a variable dependent on one or more variables.

**Results:**

There was a statistically significant improvement of all parameters investigated: total mass (− 5.68 kg), fat mass (− 4416.85 g), fat mass index (− 1724.56), visceral adipose tissue (− 332.76 g), arm circumference (− 1.63 cm) and calf circumference (− 1.16 cm). As it is reasonable to expect, even the fat free mass has been reduced (− 1236.03 g); however, the skeletal muscle index was not affected. Statistically significant improvement in glycaemic and lipid profile were reported. The BMI average reduction continued from discharge until T4. No statistically significant changes in fat free mass and visceral adipose tissue (VAT) were reported during a year of follow-up.

**Conclusions:**

The present study demonstrated the clinical benefits of 8-week MRP, which includes hypocaloric diet, physical exercise, and psychological support.

**Level of evidence:**

III, evidence obtained from cohort or case–control analytic studies.

## Introduction

Obesity is more than the simplest energy intake in excess of energy expenditure [1]. The critical issue is that obesity is a disease that generates other diseases. It is associated with greater risk of developing several chronic diseases [2–8], with consequent increased risk of disability [9]. Weight loss goal, ranging from 5 to 10% of initial body weight, is associated with a reduction in many of the health complications of obesity [10].

Lifestyle modification is considered the standard of care and the first step in obesity management [11], followed by pharmacological therapy and bariatric surgery. Lifestyle changes are always desiderable and are auspicable already with a BMI > 25 kg/m^2^. Pharmacological therapy is indicated only in case of BMI > 30 kg/m^2^ or BMI > 27 kg/m^2^ in the presence of comorbidities [12]. Bariatric surgery is indicated only in case of BMI > 40 kg/m^2^ or BMI > 35 kg/m^2^ in presence of comorbidities [13].

Since obesity is a pathology characterized by a multifactorial pathogenesis with a complex and variable clinical presentation (with cardiovascular, respiratory, osteoarticular, endocrine–metabolic and psychosocial manifestations), the therapeutic approach must be equally complex (interdisciplinary and integrated), both in the assessment phase and in the management [14].

A point of great importance is the choice of the treatment setting and the intensity of the therapeutic–rehabilitative intervention [15]. This can’t be in function of the only grade diagnosis of obesity, defined through the BMI. Rather, it must answer criteria of overall severity assessed by method of clinicians on the basis of medical comorbidity e psychiatric, disability and other factors perpetuation of the problem and risk of relapse (e.g., age, familiarity, lifestyle habits) [14]. Obesity is a chronic condition that requires continuous care, behavioural therapies and psychological support [16]. Therefore, the hospitalization, that guarantees a multidimensional approach, appears to be a successful strategy for a weight loss program.

The purpose of this study was to evaluate the effectiveness of 8-week MRP on weight loss, BMI, body composition (fat mass, fat free mass, visceral adipose tissue, skeletal muscle index) assessed by DXA and metabolic blood parameters (glycaemic and lipid profile, inflammatory status, vitamin and mineral blood values) at the beginning (T0) and at the discharge (T1).

The secondary endpoint was the evaluation of the patients' adherence to diet by BMI and body composition parameters (fat mass, fat free mass, visceral adipose tissue) during the check-up outpatient visits, at 2 (T2), 6 (T3) and 12 (T4) month follow-up after discharge.

## Methods

### Trial design and setting

This is a prospective cohort study with 1-year follow-up in which obese participants were first administered with a restrictive hypo-caloric diet under hospitalization for a maximum period of 3 months, with a range between 17 and 91 days, in the Metabolic Rehabilitation Unit of the Azienda di Servizi alla Persona, Istituto Santa Margherita, University of Pavia (27100 Pavia, Italy).

Second, this study followed up patients' adherence to diet during the check-up outpatient visits at 2 (T2), 6 (T3), and 12 (T4) months after discharge.

The study design was approved by the ethics committee of the University of Pavia, and an individual written informed consent was obtained from each participant. Data were gathered from 1 January 2016 to 1 March 2021. All the methods were performed in accordance with the CONSORT guidelines [17]. Outcomes were assessed at the beginning (T0) and at the end of the recovery period (T1) and during the outpatient visits (T2, T3, T4).

### Participants

A total of 178 subjects were enrolled in this study, 61 males and 117 females. Eligible participants were aged > 18 years with BMI ≥ 30 kg/m^2^ with one or more with metabolic comorbidities (type 2 diabetes mellitus, dyslipidemia, high blood pressure, hyperuricemia, etc.…).

### Multidimensional residential program

*Nutritional intervention* Body weight reduction was induced by a low-energy mixed diet (55% carbohydrates, 30% lipids and 15% proteins) providing 600 kcal less than individually energy requirements based on the measured TEE. The energy content and macronutrient composition of the diets adhered to the nutritional recommendations of the American Diabetes Association [18, 19]. These diets were designed to achieve weight losses of 0.5–1 kg per week; this type of diet is considered to be a low-risk intervention [20].

Individual diet plans were drawn up for each subject by the research dietitian. To optimize compliance, dietary instructions were reinforced each week by the same research dietician. Each consultation included a nutritional assessment and weighing.

Patients were administered with vitamin D supplement only if they presented a value of 25-hydroxyvitamin D (25OHD) < 30 ng/ml in blood tests at the beginning [21]. No other vitamin supplements were provided.

*Physical activity *The exercise program was based on the physical activity recommendations for adults proposed by the World Health Organization [22], on progression models in strength and aerobic training for healthy adults. Since there is limited information regarding the ideal exercise model for morbidly obese adults, we will combine strength and aerobic training (i.e., a concurrent training protocol), as previous findings in obese adults displayed important benefits when both strength and aerobic exercise are implemented in the same session [23] of 60 min of 5 days a week and more than 10,000 steps per day.

Physical activity was individualized and conducted every day by each subject with the help of qualified and properly trained physiotherapist.

*Psychological support Ps*ychological management is based on the enhanced cognitive behavior therapy (CBT-E) approach, that is considered the most valid methodology for the treatment of eating disorders [24].

Psychological support during the MRP had the dual purpose of defining the presence of eating disorder and providing psychoeducation and strategies for adhering to the new diet. Individual interviews have been carried out weekly with the aim of reducing psychopathology, if present, investigating the factors of maintenance of the disorder and carrying out a cognitive restructuring. In addition, multidisciplinary group meetings are held with an expert dietician to identify functional strategies for managing the diet once back home.

### Biochemical analysis

Blood samples were collected at baseline and at the end of the treatment. In particular, nutritional status, lipid profile, glycemic profile and status of inflammation were assessed.

Serum iron, lipids, uric acid, creatinine, and calcium were measured by enzymatic–colorimetric assay (Abbott Laboratories). PCR, Transferrin, Apo A1 and Apo B were determined by immunoturbidimetry (Roche). ESR was measured by the Westergren method using a Diesse Analyzer, blood electrolytes by indirect ISE potentiometry (Abbott Laboratories), ionized calcium by selective electrode potentiometry, and insulin by electro-chemiluminescence immuno-assay (ECLIA) (Roche Diagnostics). Blood glucose, aspartate aminotransferase (AST) and alanine aminotransferase (ALT) were analyzed by Enzymatic UV Assay (Abbott Laboratories) and CBC by differential blood cell counter. Insulin resistance was evaluated using the Homeostasis Model Assessment (HOMA) [25].

### Anthropometric measurements

Anthropometric parameters, such as body weight, waist and hip circumference were measured weekly during recovery period. Body weight was measured to the nearest 0.1 kg, using a precision scale; participants wore light clothing, no shoes, and a standardized method was used [26]. The waist was measured at the midpoint between the top of the hip bone (iliac crest) and lowest rib, using a standardized method.

### Assessment of REE

Respiratory exchange measurements using indirect calorimetry (Deltatrac Monitor II MBM-200, Datex Engstrom Division, Instruments Corp. Helsinki, Finland) were used to estimate REE, adhering to the recommended measurement conditions [27].

REE was calculated from O_2_ and CO_2_ volumes—as well as from urine excretion nitrogen values—using the Weir formula and expressed as kcal/day to obtain postprandial respiratory quotient (RQ) and substrate oxidation, continuous gas exchange was determined [28].

### Body composition assessment by double X-ray densitometry

Body composition (fat free mass, fat mass, visceral fat mass) was determined by dual-energy X-ray absorptiometry (DXA), using a Lunar Prodigy DXA (GE Medical Systems). In vivo CVs were 0.89% for whole body fat (fat mass) and 0.48% for FFM. The Skeletal Muscle Index (SMI) was taken as the sum of the fat-free soft tissue mass of arms and legs divided by height^2^. Whole body and fat free mass (FFM) were divided by height squared to obtain FFM index (FFMI). FFM depletion was defined as having whole-body FFMI below the 5th centile for age- and gender-matched healthy subjects [29]. Visceral adipose tissue (VAT) volume was estimated using a constant correction factor (0.94 g/cm^3^). The software automatically places a quadrilateral box, which represents the android region, outlined by the iliac crest and with a superior height equivalent to 20% of the distance from the top of the iliac crest to the base of the skull [30]. Subcutaneous abdominal fat was defined as the difference between android fat and visceral fat. The in vivo CVs were 0.89% and 0.48% for FM) and FFM, respectively [31].

### Statistical analysis

Statistical analysis has been carried out through the combined use of R, SPSS software with graphic integration of JASP and JAMOVI software.

The normality of the data has been verified through the Kolmogorov–Smirnov test. The data show a distribution in line with normality, and therefore, a parametric analysis of the data was applied. The descriptive characteristics of the sample was reported through the mean and standard deviation. The categorical variables were reported at the descriptive level through absolute numbers and non-cumulative percentages.

The effects of hospitalization on blood chemistry tests have been calculated from the mean and standard deviation at baseline and after hospitalization. The difference in values ​​(end of hospitalization compared to baseline) was calculated through the univariate GLM procedure, which provides regression analysis and analysis of variance for a variable dependent on one or more factors and/or variables. It was possible to adjust the data for hospitalization time and investigate the interactions between factors.

The same model was applied for anthropometric measurements. The adjusted data of the difference between means were reported together with the minimum and maximum values ​​of the 95% confidence interval. A significance level of less than 0.001 was set.

## Results

The study included a total of 178 adult patients (61 males and 117 females) with severe obesity and obesity-related comorbidities, admitted to the MRP. The data collected in the study refer to patients hospitalized from 1 January 2016 to 1 March 2021.

The anthropometric characteristics (weight, height, and BMI) of the patients at baseline are shown in Table 1. The average age of the subjects was 58.48 ± 13.97 years and the BMI was 41.30 ± 6.31 kg/m^2^. Concerning compliance to diet and physical activity during the MRP, no gender differences were observed.

**Table 1:** Anthropometric characteristics at baseline

Variable	Mean value ± ds
Subjects *n* = 178 (M:61; F:117)	
Age (years)	
Total	58.48 ± 13.97
Male	55.08 ± 14.97
Female	61.78 ± 12.89
Height (m)	
Total	1.60 ± 0.11
Male	1.70 ± 0.07
Female	1.54 ± 0.07
Weight (kg)	
Total	106.28 ± 20.68
Male	120.50 ± 18.43
Female	99.10 ± 17.85
BMI (kg/m^2^)	
Total	41.30 ± 6.31
Male	41.3 ± 6.72
Female	41.4 ± 5.52

### Effect of rehabilitation hospitalization on blood chemistry parameters

The blood chemistry parameters at the beginning and at the end of hospitalization are shown in Table 2. There was a statistically significant improvement (*p* < 0.001) in the glucose profile, with a reduction in blood glucose, glycated haemoglobin, insulin and HOMA index values.

**Table 2:** Blood chemistry parameters at the beginning and at the end of the treatment

Variable	Pre (mean ± ds)	Post (mean ± ds)	Δ change (CI: lower; upper)	*P* value
Folate	6.92 ± 5.44	12.03 ± 8.64	5.11 (3.10; 7.12)	< 0.001*
Vit B12	355.11 ± 143.46	384.15 ± 141.80	29.04 (10.31; 47.76)	0.003
Fe	85.32 ± 31.16	70.8 ± 21.86	– 14.53 (– 19.13; – 9.92)	< 0.001*
Transferrin	260.19 ± 52.62	236.05 ± 45.86	– 24.14 (– 29.71; – 18.58)	< 0.001*
Vit D	18.88 ± 11.62	32.22 ± 14.43	13.34 (10.35; 16.34)	< 0.001*
ESR	24.24 ± 18.82	24.48 ± 17.92	0.236 (– 2.16; 2.63)	0.846
CRP	0.69 ± 0.83	0.44 ± 0.42	– 0.25 (– 0.38; – 0.12)	< 0.001*
Glycemia	97.93 ± 22.12	87.83 ± 13.84	– 10.10 (– 12.48; – 7.72)	< 0.001*
Hb1Ac	6.82 ± 1.33	6.22 ± 0.88	– 0.59 (– 0.80; – 0.37)	< 0.001*
Insulin	15.11 ± 8.02	12.46 ± 7.30	– 2.64 (– 3.78; – 1.51)	< 0.001*
HOMA	3.74 ± 2.46	2.71 ± 1.80	– 1.03 (– 1.41; – 0.65)	< 0.001*
Uricemia	6.50 ± 1.49	6.42 ± 1.64	– 0.07 (– 0.29; 0.14)	0.508
Azotemia	39.94 ± 15.64	40.24 ± 15.9	0.30 (– 1.52; 2.12)	0.744
Creatinine	0.89 ± 0.25	0.94 ± 0.32	0.05 (0.03; 0.08)	< 0.001*
Na	139.4 ± 2.09	140.03 ± 1.87	0.62 (0.25; 0.99)	0.001
K	4.41 ± 0.42	4.39 ± 0.40	– 0.02 (– 0.08; 0.04)	0.457
Cl	103.58 ± 3.03	104.16 ± 2.85	0.058 (0.13; 1.03)	0.012
Ca	9.360 ± 0.49	9.43 ± 0.46	0.13 (0.06; 0.20)	< 0.001*
Chol Tot	184.78 ± 40.38	160.49 ± 32.86	– 24.35 (– 28.93; – 19.77)	< 0.001*
HDL	45.48 ± 11.74	40.81 ± 8.90	– 4.67 (– 5.80; – 3.54)	< 0.001*
TRG	141.92 ± 62.40	118.65 ± 43.17	– 23.27 (– 29.35; – 17.19)	< 0.001*
LDL	111.93 ± 37.35	99.01 ± 33.48	– 12.92 (– 17.53; – 8.31)	< 0.001*
Apo A	134.04 ± 25.62	119.28 ± 18.77	– 14.72 (– 17.51; – 12.02)	< 0.001*
Apo B	100.75 ± 25.82	87.82 ± 22.03	– 12.93 (– 15.91; – 9.95)	< 0.001*
AST	19.90 ± 7.15	19.55 ± 6.55	– 0.35 (– 1.26; 0.57)	0.457
ALT	25.61 ± 13.35	26.01 ± 12.64	0.41 (– 1.32; 2.13)	0.642
gGT	31.05 ± 21.63	22.07 ± 12.89	– 8.98 (– 11.17; – 6.79)	< 0.001*
Prealb	24.20 ± 5.00	22.49 ± 4.55	– 1.74 (– 2.29; – 1.19)	< 0.001*
Phosphatase	63.29 ± 20.63	58.80 ± 18.74	– 4.49 (– 6.40; – 2.57)	< 0.001*
Bilir tot	0.71 ± 0.29	0.58 ± 0.25	– 0.13 (– 0.16; – 0.10)	< 0.001*
Cholinesterase	10,394.85 ± 2010.58	92.77 ± 1924.63	– 1117.29 (– 1310.45; – 924.12)	< 0.001*
Lipase	23.85 ± 12.57	26.42 ± 12.70	2.57 (0.90; 4.23)	0.003
Amylase	50.30 ± 17.52	54.62 ± 19.30	4.32 (2.29; 6.34)	< 0.001*
Homocysteine	17.93 ± 5.61	16.12 ± 4.87	– 1.81 (– 2.80; – 0.81)	< 0.001*
TSH	1.97 ± 1.26	1.98 ± 1.68	0.01 (– 0.23; 0.25)	0.916
Alfa 2%	10.38 ± 1.72	10.07 ± 1.72	– 0.30 (– 0.47; – 0.13)	0.001
Beta %	11.99 ± 1.50	11.50 ± 1.45	– 0.50 (– 0.63; – 0.37)	< 0.001*
Gamma %	15.28 ± 2.98	15.16 ± 2.89	– 0.12 (– 0.35; 0.11)	0.294
WBC	7.01 ± 1.80	6.31 ± 1.76	– 0.70 (– 0.88; – 0.52)	< 0.001*
Linf n	2.345 ± 0.77	2.26 ± 0.71	– 0.08 (– 0.16; 0.01)	0.030
Linf %	32.96 ± 7.03	36.24 ± 7.65	3.32 (2.44; 4.20)	< 0.001*
RBC	4.73 ± 0.50	4.65 ± 0.47	– 0.08 (– 0.12; – 0.04)	< 0.001*
HGB	13.47 ± 1.40	13.28 ± 1.30	– 0.19 (– 0.31; – 0.07)	0.002
HCT	41.19 ± 3.93	40.65 ± 3.70	– 0.53 (– 0.93; – 0.14)	0.008
MCV	87.89 ± 5.11	88.27 ± 4.64	0.38 (0.04; 0.072)	0.030
PLT	252.02 ± 61.46	230.25 ± 56.57	– 21.77 (– 26.95; – 16.59)	< 0.001*

95% Confidence Interval of the Difference

*Statistically significant

It also improved the lipid profile with a statistically significant reduction (*p* < 0.001) in total cholesterol, LDL cholesterol and triglycerides levels. However, HDL cholesterol was significantly reduced, too.

Regarding the blood values ​​of minerals and vitamins, it was reported statistically significant increase (*p* < 0.001) in the levels of folate, vitamin B12, vitamin D and calcium. Furthermore, a statistically significant reduction (*p* < 0.001) in iron and transferrin levels was observed.

### Effect of rehabilitation hospitalization on anthropometric parameters

The anthropometric and body composition parameters at the beginning and at the end of hospitalization are shown in Table 3. It was reported a statistically significant reduction (*p* < 0.001) of all the parameters investigated, with the exception of the skeletal muscle index (SMI), which was reduced, but not in a statistically significant manner.

**Table 3:** Body composition parameters at the beginning and end of the treatment

Variable	Pre (mean ± ds)	Post (mean ± ds)	Δ change (CI: lower; upper)	*P* value
Arm (cm)	36.82 ± 4.62	35.19 ± 4.06	– 1.63 (– 1.96; – 1.31)	< 0.001*
Calf (cm)	41.96 ± 4.25	40.80 ± 4.06	– 1.16 (– 1.39; – 0.93)	< 0.001*
Total mass (kg)	104.02 ± 18.61	98.35 ± 17.20	– 5.68 (– 6.24; – 5.11)	< 0.001*
FFM (g)	51,705.83 ± 10,503.63	50,469.80 ± 9609.47	– 1236.03 (– 1595.49; – 876.57)	< 0.001*
FM (g)	49,292.27 ± 10,768.96	44,875.43 ± 10,346.06	– 4416.85 (– 4847.30; – 3986.39)	< 0.001*
FM (%)	48.91 ± 5.79	46.96 ± 5.98	– 1.96 (– 2.26; – 1.65)	< 0.001*
FFMI	20,124.69 ± 2536.29	19,537.83 ± 2753.25	– 586.86 (– 913.19; – 260.52)	< 0.001*
FMI	19,481.78 ± 4607.48	17,757.22 + 4465.56	– 1724.56 (– 1885.48; – 1563.64)	< 0.001*
VAT	2389.23 ± 1028.66	2056.48 ± 894.83	– 332.76 (– 412.79; – 252.72)	< 0.001*
SMI	9.46 ± 1.17	9.30 ± 1.19	– 0.157 (– 0.26; – 0.05)	0.003

95% Confidence Interval of the Difference

*FFM*, fat free mass, *FM* fat mass, *FFMI* fat free mass index, *FMI* fat mass index, *VAT* visceral adipose tissue

*Statistically significant

**Fig. 1:**
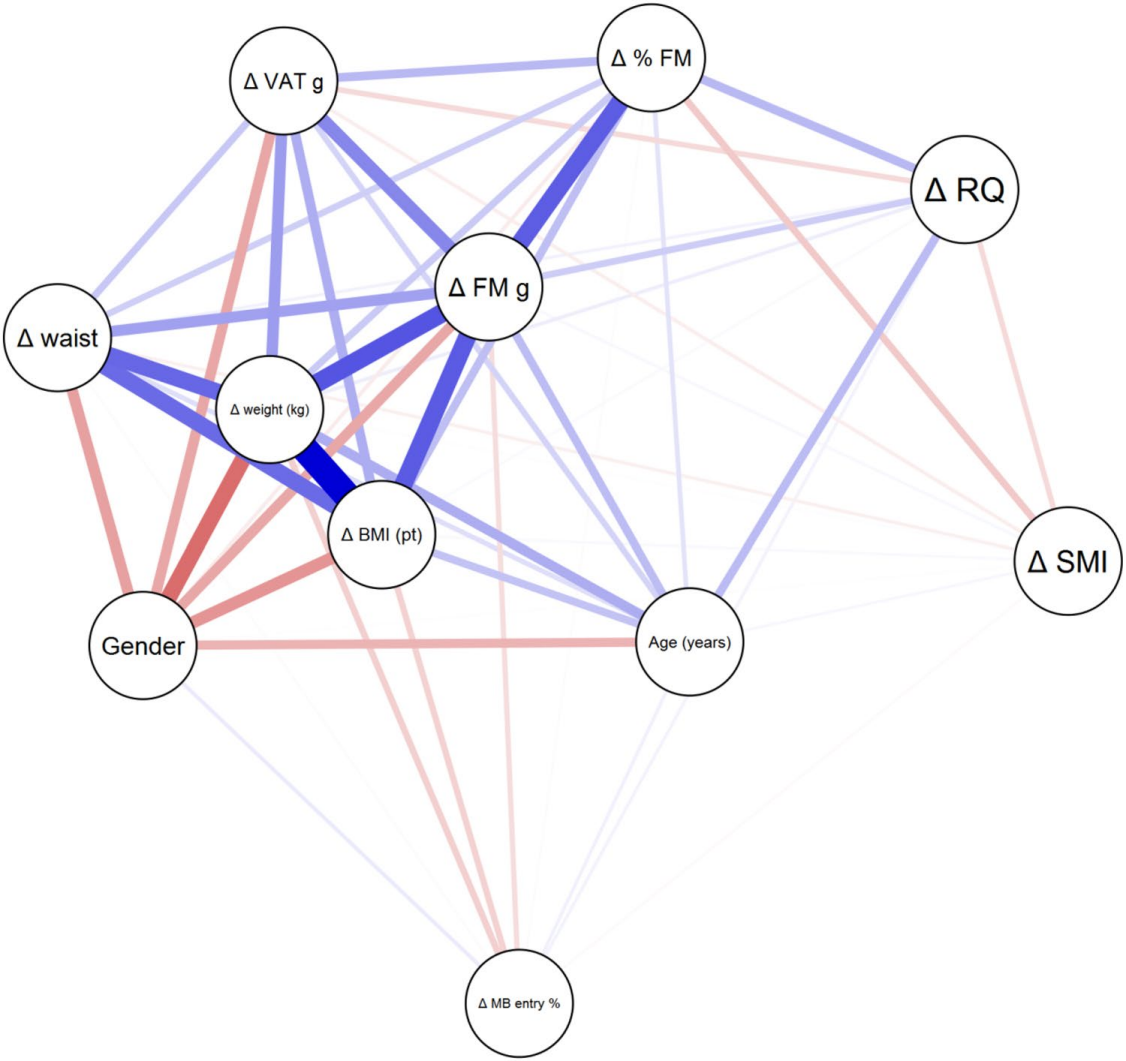
Body composition network analysis

In Fig. 1, the correlations between the different factors investigated are reported. Specifically, the blue lines represented the positive correlations, while the red lines represented the negative ones.

For example, a strong positive correlation was observed between the change (decrease) in weight and changes in BMI, waist circumference, fat mass (expressed in grams and in percentage). The latter, in turn, was positively associated with a reduction in visceral adipose tissue.

It is interesting to note that weight loss was no way linked to the reduction of SMI, to signify how the weight loss detected was, instead, due to the loss of adipose mass.

**Fig. 2:**
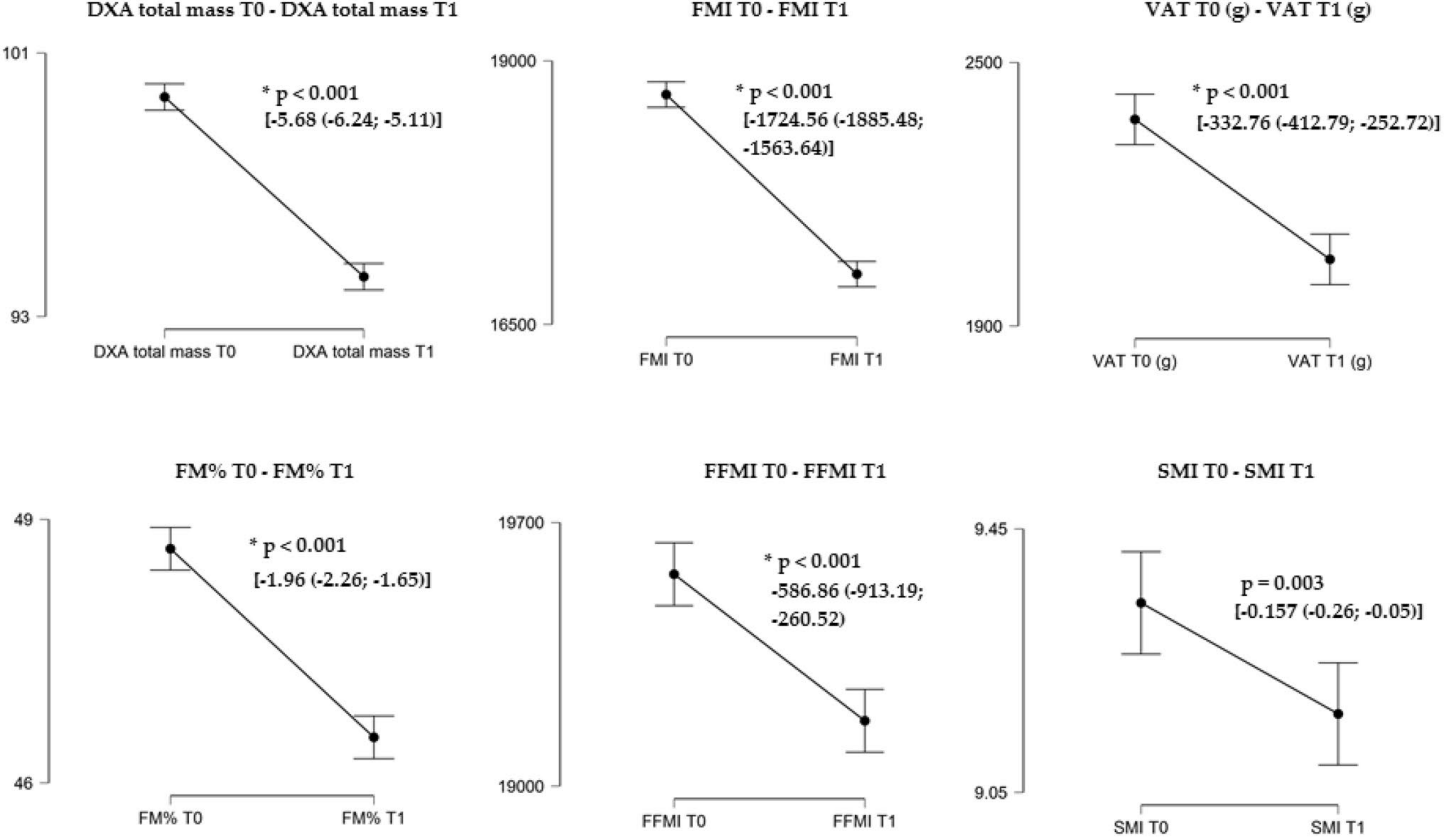
Changes in body composition during the hospitalization period

A marked red line was observed; therefore, a strong negative correlation, between weight loss and gender: when gender moved from man to woman, the weight loss is lower. Modifications in body composition during the hospitalization period are reported in Fig. 2.

### Changes in body composition for 1-year follow-up

The trend of the main anthropometric parameters and those related to body composition (weight, BMI, lean mass, fat mass and visceral adipose tissue) from discharge to the follow-up visits were investigated. Specifically, T2, T3, T4 corresponded to control visit at 2, 6, and 12 months after discharge, respectively.

**Fig. 3:**
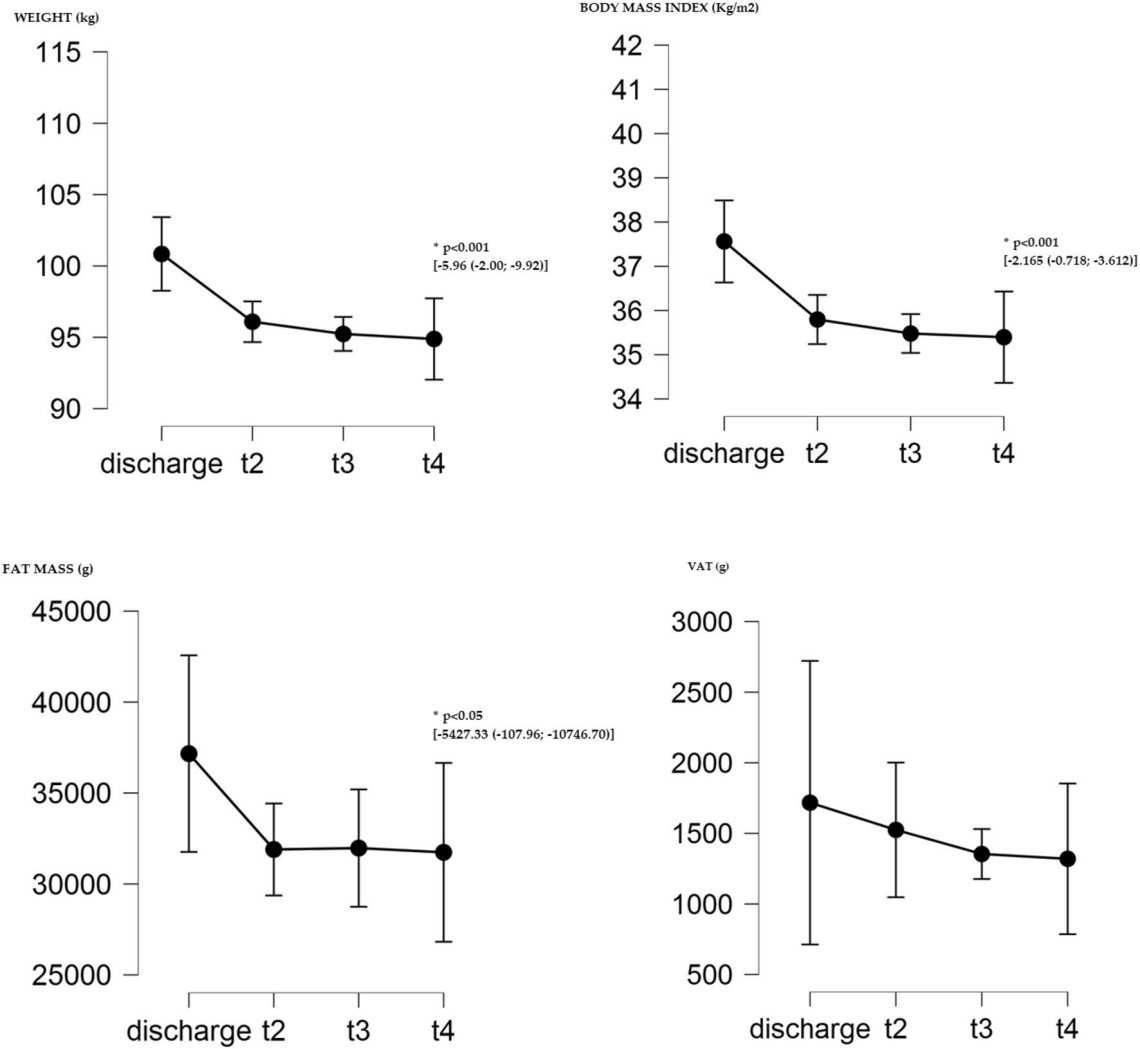
Changes in body weight, body mass index, fat mass, and VAT for 1-year follow-up

The average reduction of body weight and BMI continued from discharge until T4, but these changes were statistically significant (*p* < 0.001) only at T4. Even the average loss of fat mass, continued from discharge up to T4, and this reduction was statistically significant (*p* < 0.05) only at T4. In this case, the significance value was set as *p* < 0.05. No statistically significant changes in fat free mass and VAT were reported (see Table 4; Fig. 3).

**Table 4:** Variations from discharge up to 1 year of follow-up

Time	Mean value	Mean difference	Lower	Upper	P value
Body weight (kg)					
Discharge	100.84				
T2	96.09	– 4.75	– 0.79	– 8.71	0.011
T3	95.24	– 5.60	– 1.64	– 9.57	0.002
T4	94.88	– 5.96	– 2.00	– 9.92	< 0.001*
Body mass index (kg/m^2^)					
Discharge	37.56				
T2	35.80	– 1.77	– 0.32	– 3.21	0.009
T3	35.48	– 2.08	– 0.64	– 3.53	0.001
T4	35.40	– 2.17	– 0.72	– 3.61	< 0.001*
Fat mass (g)					
Discharge	37,165.33				
T2	31,896.33	– 5269.00	50.37	– 10,588.37	0.052
T3	31,972.67	– 5192.67	126.70	– 10,512.04	0.056
T4	31,738.00	– 5427.33	– 107.96	– 10,746.70	< 0.05*
Fat free mass (g)					
Discharge	45,072.33				
T2	44,149.00	– 923.33	2285.30	– 4131.97	1.000
T3	43,436.67	– 1635.67	1572.97	– 4844.30	0.579
T4	44,174.00	– 898.33	2310.30	– 4106.97	1.000
Visceral adipose tissue (g)					
Discharge	1717.33				
T2	1524.67	– 192.67	598.19	– 983.52	1.000
T3	1354.00	– 363.33	427.52	– 1154.19	0.758
T4	1319.67	– 397.67	393.19	– 1188.52	0.601

*Statistically significant

## Discussion

The results of the study have showed the efficacy of MRP for the treatment of obesity in terms of weight loss and body composition. In addition, improvements in blood chemistry were reported at the end of the weeks of hospitalization. Regarding compliance to diet and physical activity during the MRP, no gender differences were observed.

As regards the anthropometric and body composition parameters, the results obtained have revealed a significant statistical improvement of all the parameters investigated: total mass, fat mass, fat mass index, visceral adipose tissue, arm and calf circumferences. As it is reasonable to expect, even the fat free mass has been reduced; however, the SMI was not affected. This data was positive as it reflects the fact that weight loss occurred correctly: weight reduction has mainly affected fat mass, while lean mass was preserved.

Concerning the blood chemistry parameters, there was a statistically significant improvement in the glycaemic profile with a reduction in glycaemic values, glycated haemoglobin, insulin and HOMA index. It also improved the lipid profile with a statistically significant reduction of total cholesterol values, LDL cholesterol and triglycerides. Finally, there was an improvement in the levels of folate, vitamin B12 and calcium.

Lifestyle changes are at the pivot of any obesity treatment program, but these may be difficult to maintain in real life, where personal and social factors can hinder patients' efforts to change. MRP for the treatment of obesity are widely used for the management of obesity and have proven to be more effective than outpatient programs, at least in the short term [32]. Although less frequent, hospitalizations for the treatment of obesity in adults can also lead to important results.

The results of the present study are in agreement with the results of the study by Budui et al. which concluded that 3 weeks of a MRP led to significant clinical and functional improvements, similarly in young and elderly patients suffering from severe obesity [9]. In the long term, these improvements are reflected in a better quality of life, through better management of comorbidities associated with obesity, and a reduction in the state of frailty [9]. Moreover, Capodaglio et al., showed that a 4-week MRP was effective in reducing moderate and severe disabilities of obesity patients with orthopaedic comorbidities and improves functional abilities [33]. However, changes in body weight do not appear to be related to changes in disability test scores. This suggests that other factors besides body weight have an impact on functional improvements [33].

The improvement in body composition in subjects with obesity of both sexes, was confirmed even by Maffiuletti et al. after a 3-week hospitalization, based on similar intervention based on nutritional therapy with calorie restriction and nutritional education, physical activity and psychological counseling [34]. According to Haslacher et al., a 3-week rehabilitation hospitalization leads to a reduction not only in body weight, but also in cardiovascular risk of 30–35% calculated by the Framingham HARD CHD score [35]. The values ​​of c-reactive protein, lipid and carbohydrate metabolism and liver function also improve, so weight loss also reflects a decrease in the inflammatory state linked to obesity [35].

Second, the results obtained in the present study have revealed that average reduction of body weight, BMI and fat mass continued from discharge until T4; a reduction of VAT was detected, but the change was not statistically significant. Moreover, no statistically significant changes in fat free mass and VAT were reported during 1 year of follow-up. Similarly, the medium-term effects of the 3-week recovery were assessed by Maffiuletti et al.: 11 months after hospitalization, 75% of patients managed to maintain a body weight lower than baseline. Clinical success at follow-up was associated with higher levels of reported physical activity than in those who regained weight; as a result, subjects who continued to lose weight had greater muscle mass and strength and reduced cardiovascular risk factors (lower total cholesterol and glycemic levels, and higher HDL cholesterol) than the others [34].

Otherwise, the results obtained by Tadokoro et al. during the follow-up reveal that the BMI remained unchanged 3 months after discharge, but increases modestly at 1 year after discharge, regardless of the weight lost during hospitalization [36]. The authors, have investigated the factors involved in weight loss and its maintenance in patients suffering from morbid obesity [36]. The excess weight lost during the weeks of hospitalization is not correlated with its maintenance during the follow-up [36]. The presence of diabetes does not affect the amount of weight lost during hospitalization. However, diabetic patients show less body weight gain after discharge, possibly due to the effects of antidiabetic drugs [36].

The benefits of rehabilitation hospitalization are expressed not only on a physical level, but also on the mental state of the patient suffering from obesity [37]. Subsequently, in 2 years of follow-up, patients should implement a permanent lifestyle change by improving their diet and fighting a sedentary lifestyle [37].

Buscemi et al., have investigated the psychological and behavioural factors associated with long-term weight maintenance after a MRP for the treatment of obesity, concluding that the psychological quality of life is associated even with modest amounts of weight loss in the long run [38].

The relevance of a multidisciplinary approach to obesity is stressed in the study by Donini et, in which two groups of obese patients are compared, one following a standard dietetic treatment and the other following a multidisciplinary program, based on nutritional plan, physical reconditioning and cognitive–behavioural psychotherapy [39]. The latter achieved better weight loss, better physical performance and also the scores on the tests regarding anxiety, mood and quality of life improved [39].

A multidimensional multidisciplinary approach based on nutritional intervention and psycho-physical rehabilitation, set against a conventional dietetic therapy, was more effective in the long-term outcome of obesity with regard to weight loss, physical activity, possible eating disorders, and obesity-related complications [40].

The importance of continuous intervention by physicians, dieticians and clinical psychologists was emphasized in a previous work, in which patients who follow biweekly instructions for a year significantly maintained body weight loss, with a slight weight gain of 0.4 kg, unlike those who didn’t follow the directions and registered an increase of 5.1 kg [41].

Furthermore, successfully achieving a good weight loss goal during the rehabilitation program involves maintaining a lower weight afterwards without increasing the risk of dropping out. Satisfactory weight loss during hospitalization could increase the motivation of patients suffering from obesity [42].

Some nutritional treatment fails due to patients' poor compliance with the prescribed program. For this reason, constant monitoring and psychological support are necessary.

The strength of this study was given, first of all, by the sample size. Second, not only was a comparison of the clinical status of the patients at the beginning and at the end of the treatment carried out, but the subjects were also evaluated during a 1-year follow-up.

The main limitation of the study was the absence of a control group. Further investigations will be precisely to compare the group of obese patients who have undergone hospitalization with another group of obese patients followed only on an outpatient basis, in terms of weight loss and adherence in the short and long term to the proposed nutritional treatment.

## Conclusions

The present study demonstrated the clinical benefits of 8-week MRP, which includes hypocaloric diet, exercise and psychological support in patients affected by obesity. Considering the complex clinical status and comorbidities of these patients, a multidisciplinary residential setting represents an optimal setting for the management of obesity.

### What is already known on this subject?

It’s well known that obese patients need a multidimensional approach. In literature there are some studies that have demonstrated the efficacy of the hospitalization, that guarantees a multidimensional approach, and it appears to be a successful strategy for a weight loss program.

### What does this study add?

The present study not only investigates the trend of obese patients during the 8-weeks MRP, but also their trend from discharge up to 1 year of follow-up. Moreover, the novelty of this study is the use of the DXA for the assessment of body composition, while the existing studies on MRP often use bioelectrical impedance, and even the VAT with its important clinical meaning, measured during hospitalization, then at 2, 6, and 12 months after discharge. In clinical practice it is important to evaluate the changes in body composition that occur during a weight loss program and how these are maintained over the follow-up period.
